# 4516 Sex Differences in the Cortical Structure in Children with Irritability and Disruptive Behavior

**DOI:** 10.1017/cts.2020.426

**Published:** 2020-07-29

**Authors:** Karim Ibrahim, Fangyong Li, George He, Kevin Pelphrey, Gregory McCarthy, Denis Sukhodolsky

**Affiliations:** 1 Yale University School of Medicine; 2 Yale University; 3 University of Virginia

## Abstract

OBJECTIVES/GOALS: This study examines sex differences in brain structure in youths with disruptive behavior disorders (DBD). We use measures of gray matter volume (GMV) in regions-of-interest implicated in the pathophysiology of conduct problems and a whole-brain analysis of cortical thickness. We also examine unique associations between brain structure and callous-unemotional traits. METHODS/STUDY POPULATION: This study included 90 children with a DBD (30 females) aged 8-16 and 50 Healthy Controls (20 females) matched for age and IQ. Children received a diagnostic evaluation using the K-SADS. Pre-processing was conducted using FreeSurfer. For ROI analyses, multivariate GLM models were conducted in SPSS for estimates of GMV to examine the main effects of diagnosis and sex, and sex-by-diagnosis interactions. Whole-brain analyses were conducted in FreeSurfer. Associations were examined between structure and parent ratings of callous-unemotional (CU) traits using the Inventory of Callous-Unemotional Traits in regression analyses in the DBD group, while controlling for the variance in aggressive behavior using the Child Behavior Checklist Aggressive Behavior Scale. All analyses controlled for differences in intracranial volume. RESULTS/ANTICIPATED RESULTS: Relative to controls, children with DBD showed reduced GMV in the bilateral amygdala (left: *p* = .004; right: *p* = .04). Sex-by-diagnosis interactions were observed in the left ventromedial prefrontal cortex (*p* = .004), right insula (*p* = .001), right inferior frontal gyrus (*p* = .02), and bilateral anterior cingulate (left: *p* = .02; right: *p* = .01) in which DBD males showed lower and DBD females showed higher GMV relative to respective controls. For whole-brain analyses, a significant sex-by-diagnosis interaction was observed in the left ventromedial prefrontal cortex and supramarginal gyrus indicating that DBD males showed lower and DBD females showed higher cortical thickness relative to respective controls. Sex-by-CU traits interactions were observed for left amygdala and ACC volumes. DISCUSSION/SIGNIFICANCE OF IMPACT: The current study provides evidence of reduced amygdala volume in children with DBD, and interactions between sex and diagnosis in the ventromedial prefrontal cortex and supramarginal gyrus, which may have implications for identifying sex-sensitive neural biomarkers. CONFLICT OF INTEREST DESCRIPTION: Disclosures: Dr. Sukhodolsky receives royalties from Guilford Press for a treatment manual on CBT for anger and aggression in children. Drs. Ibrahim, He, Pelphrey, McCarthy, and Mr. Li have no biomedical financial interests or potential conflicts of interest to declare related to this present study.

